# Next-Generation Sequencing Analysis of CpG Methylation of a Tumor Suppressor Gene SHP-1 Promoter in Stable Cell Lines and HCV-Positive Patients

**DOI:** 10.3390/v14112352

**Published:** 2022-10-26

**Authors:** Priya Devi, Katarina Engdahl, Tanel Punga, Anders Bergqvist

**Affiliations:** 1 Department of Medical Sciences, Uppsala University, SE 75185 Uppsala, Sweden; 2 Department of Medical Biochemistry and Microbiology, Uppsala University, SE 75123 Uppsala, Sweden; 3 Clinical Microbiology and Hospital Infection Control, Uppsala University Hospital, SE 75185 Uppsala, Sweden

**Keywords:** HCV, SHP-1, CpG methylation, next-generation sequencing

## Abstract

Hepatitis C virus (HCV) is the major causative pathogen associated with hepatocellular carcinoma and liver cirrhosis. The main virion component, the Core (C) protein, is involved in multiple aspects of HCV pathology including oncogenesis and immune evasion. In this study, we established a next-generation bisulfite sequencing (NGS-BS) protocol to analyze the CpG methylation profile at the tumor suppressor gene SHP-1 P2 promoter as a model system. Our data show that HCV C protein expression in the immortalized T cells correlated with a specific CpG methylation profile at the SHP-1 P2. The NGS-BS on HCV-positive (HCV^+^) patient-derived PBMCs revealed a considerably different CpG methylation profile compared to the HCV C protein immortalized T cells. Notably, the CpG methylation profile was very similar in healthy and HCV^+^ PBMCs, suggesting that the SHP-1 P2 CpG methylation profile is not altered in the HCV^+^ individuals. Collectively, the NGS-BS is a highly sensitive method that can be used to quantitatively characterize the CpG methylation status at the level of individual CpG position and also allows the characterization of cis-acting effects on epigenetic regulation.

## 1. Introduction

Epigenetics is the alteration of gene expression without any changes in the DNA sequences [1]. DNA methylation is one of the most studied epigenetic changes that involve the addition of a methyl group to a 5′-carbon of the pyrimidine ring in cytosine nucleotide by DNA methyltransferases (DNMTs) [2]. Transfer of the methyl group occurs at the cytosine residue that is followed by a guanine (CpG). Multiple CpG dinucleotides are usually detected at the gene promoter regions where they form so-called CpG islands. Since promoter CpG islands frequently overlap with gene regulatory elements, they have the the ability to control gene expression [3]. Apart from basal gene expression regulation, DNA methylation has an important role in development, genomic imprinting, X-chromosome inactivation and transposon silencing [4,5]. Notably, various virus infections induce epigenetic alterations in the infected cells. This is achieved by direct interaction of the viral proteins with various cellular factors, including the proteins involved in CpG methylation [6].

Hepatitis C virus (HCV) is one of the main causes of liver inflammation in humans. HCV can cause long-term persistent infections, which may ultimately progress to liver (hepatocellular carcinoma) and non-liver (lymphoma) cancers [7,8]. Hence, HCV is regarded as one of the oncoviruses [9]. In addition to hepatocytes, HCV infection has also been demonstrated in various extrahepatic cells including PBMCs, monocytes, macrophages, dendritic cells, T and B lymphocytes [10,11,12,13,14,15]. In lymphocytes, HCV infections have been shown by the detection of HCV negative strand RNA [16], the NS3, NS5A and core proteins [16,17,18], and the release of HCV virions by electron microscopy and verification by buoyant density [16,19,20]. The multifunctional HCV Core (C) protein has oncogenic properties due to its ability to interact with different signaling cascades in the cell [21]. A common way for the oncoviruses to cause cell transformation is by inducing hypermethylation of the tumor suppressor genes or hypomethylation of the proto-oncogenes [22]. The tumor suppressor protein, non-receptor Src homology region 2 (SH2) domain containing protein tyrosine phosphatase (SHP-1, also known as PTPN6, SHP, SHPTP-1, HCP, PTP1C) is a regulator of several signaling cascades that control cell proliferation, differentiation and inflammation. A particular feature of the human SHP-1 gene is that it can use two different promoters, P1 and P2. The P1 promoter initiates transcription from the exon 1 and is active in the epithelial cells, whereas the P2 promoter initiates transcription from the exon 2 and is most active in the hematopoietic cells [23,24,25]. In human hepatocellular carcinoma (HCC), the expression of SHP-1 is reduced at the mRNA and protein level [26,27]. In hematological malignancies, the loss or decreased expression of SHP-1 due to CpG hypermethylation in the P2 has been reported [28,29,30,31,32].

The most commonly used techniques to measure the locus specific methylation are bisulfite sequencing (BS-seq), methylation-based PCR and pyrosequencing [33]. These methods are based on the modification of a cytosine base to uracil on unmethylated cytosine by bisulfite treatment, whereas the methylated cytosine remains intact after the same treatment. The converted uracil base is amplified as thymine in the subsequent PCR step and the base difference of C/T or G/A as the readout in sequencing [34]. However, the application of aforementioned methods for CpG methylation profiling in the clinical settings is limited due to the low quantitative accuracy and lack of high-throughput sample processing [33].

**Figure 1:**
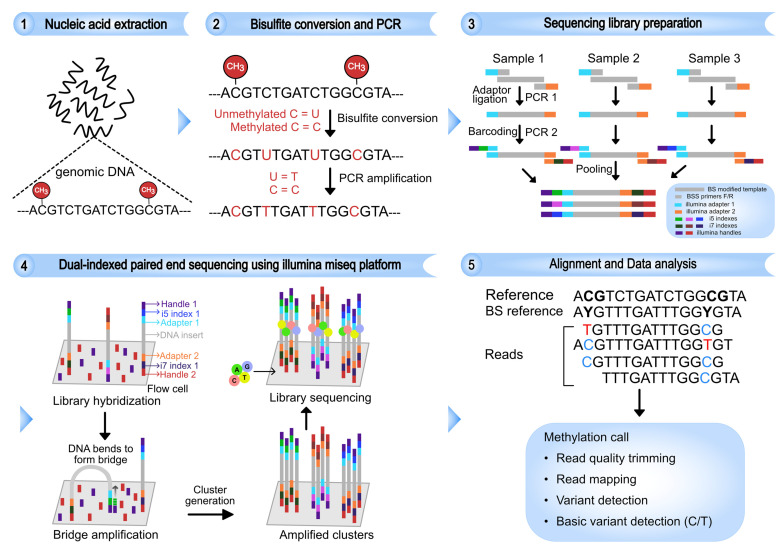
Schematic layout of the next-generation bisulfite sequencing (NGS-BS). The gDNA is isolated and modified by bisulfite (BS) treatment, which changes the unmethylated cytosine to uracil while previously methylated cytosine remains unchanged. The converted uracils are amplified as thymine in the PCR step. To simplify the sequencing library generation, the adapter sequences are tagged with the bisulfite-specific primers and the region of interest is amplified by inner PCR (PCR 1). The amplicon generated is barcoded with dual indexes in the outer PCR step (PCR 2), and samples are pooled for multiplexing. The multiplexed libraries are run on next generation sequencer (NGS), and the sequencing reads are aligned to bisulfite reference sequence. The cytosine methylation at each individual CpG position was determined, and methylation percentage is calculated.

In this study, we established a next-generation bisulfite sequencing (NGS-BS) method to analyze the CpG methylation profile at the SHP-1 P2 as a model. The workflow for the NGS-BS method is summarized in the Figure 1. Compared to BS-seq, NGS-BS offered both better performance and the ability to characterize cis-acting effects of CpG methylation. Application of the NGS-BS on HCV^+^ patient-derived PBMCs revealed a considerably different CpG methylation profile compared to the HCV C protein expressing cells.

## 2. Materials and Methods

### 2.1. Patient Samples and Cell Lines

Blood samples were collected from 11 healthy individuals and 12 patients positive for HCV RNA and anti-HCV antibodies (HCV^+^) at Uppsala University Hospital, Sweden. All subjects gave their informed consent for inclusion before they participated in the study. The study was conducted in accordance with the Declaration of Helsinki, and the protocol was approved by the regional Ethics Committee of Uppsala (Dnr 2016/238). The human Jurkat T cell and three HCV C proteins expressing Jurkat T cell line JHC.d, JHC.g and JHC.h have been characterized before [35] and were cultured in RPMI-1640 media (Sigma-Aldrich, Stockholm, Sweden) supplemented with 10% fetal calf serum and 1% penicillin–streptomycin mixture (Sigma-Aldrich) at 37 °C in a 5% CO_2_ incubator.

### 2.2. PBMC Isolation

The patient’s whole blood was diluted with equal volumes of PBS, and Peripheral Blood Mononuclear Cells (PBMCs) were separated from the blood by centrifugation on a Histopaque^TM^-1077 cushion (Thermo Fisher Scientific, Uppsala, Sweden). PBMCs from the plasma/Histopaque interface were carefully transferred to the separate tube with the help of a transfer pipette. Mononuclear cells were washed four times with PBS and stored at −80 °C for later usage.

### 2.3. Strand-Specific Quantitative RT PCR (ssqRT-PCR) Recognizing HCV RNA

The RNA template was denatured at 70 °C for 5 min to degrade the secondary structures if present and placed on ice for 2 min. For the specific detection of (+) and (−) RNA, reverse transcription (RT) was performed with tagged primers HCV_R tag T(5′-GGCAGTATCGTGAATTCGATGCTCCAAGAAAGGACCCRGTC-3′) and HCV_F tag T (5′-GGCAGTATCGTGAATTCGATGCTCCCGGGAGAGCCATAGTG-3′) primers, respectively, at 50 °C for 30 min, and the enzyme was inactivated at 85 °C for 5 min. A non-viral tag sequence (underlined) was added to the 5′ end of the respective RT primers to avoid unspecific priming. The RT reaction contained the Invitrogen reagents 5× buffer, 5 mM DTT, 0.5 mM dNTPs, 100 nM RT primers 40 U RNaseOut and 1 U of Superscript III Reverse Transcriptase (Thermo Fisher Scientific). Unincorporated primers and dNTPs in the RT mixture were removed by treatment with 2 µL Illustra ExoProStar 1-step kit (GE Healthcare, Uppsala, Sweden) at 37 °C for 15 min, inactivated by heating at 85 °C for 15 min and then diluted to 200 µL (1:10) with nuclease-free water.

### 2.4. Real-Time HCV PCR (qRT-PCR)

The detection of (+) RNA was performed with HCV_F flap primer, which is complementary to the HCV positive sense strand (+), and the detection of (−) RNA was performed with HCV_R flap primer complementary to the HCV negative sense (−) strand in quantitative PCR (qPCR). Subsequently, the (+) RNA was amplified by HCV_tag T flap (5′-aataaatcataaGGCAGTATCGTGAATTCGATGC) reverse primer and HCV_F flap (5′-aataaatcataaTCCCGGGAGAGCCATAGTG-3′) as the forward primer. Conversely, (−) RNA was amplified by HCV_Tag T flap (5′-aataaatcataaGGCAGTATCGTGAATTCGATGC) primer as forward primer and HCV_R flap (5′-aataaatcataaTCCAAGAAAGGACCCRGTC-3′) as reverse primer. The purpose of adding a 12 nucleotide AT-rich flap sequence (lowercase letters) to the 5′ end of the primers was to increase the fluorescent signal strength [36]. For detection, the probe 5′-FAM-TCTKCGGAACCGCTG-MGB-3′ was used. The qRT-PCR reaction was carried out on a Rotor-Gene 3000 PCR system (Corbett Research, Mortlake, Australia) and analyzed using Rotor-Gene Q series software version 6.1.93 (Qiagen, Sollentuna, Sweden). qRT-PCR reaction was prepared using Applied Biosystems TaqMan™ Universal PCR Master Mix (Thermo Fisher Scientific) containing 5 µL of cDNA, 2× Taqman mastermix, and final concentrations for primers and probe were 400 nM and 200 nM, respectively. The template was amplified at 95 °C for 10 min; 95 °C for 15 s; 57 °C for 60 s; for 50 cycles. Total HCV RNA from plasma and PBMCs were analyzed on Cobas Ampliprep/Cobas Taqman HCV assay (CAP-CTM; Roche Diagnostics, Solna, Sweden) and Abbott RealTime HCV m2000 assay (ART; Abbott Scandinavia, Solna, Sweden).

### 2.5. PBMC Cell Type Analysis by Flow Cytometry

The phenotypic characteristics of PBMCs were analyzed by staining surface antigens with specific antibodies (Beckman Coulter, Solna, Sweden). Briefly, 50 µL of cell suspension was mixed with 2 µL of antibody solution and incubated for 15 min in the dark. The antibodies were directly conjugated to following fluorochromes: CD14-FITC, CD19-PC5.5, CD16-PC7, CD56-PC7, CD2-APC-AF750 and CD45-Krome orange in 250 µL optilyse C lysis solution (Beckman Coulter) and incubated for 15 min in the dark. To minimize autofluorescence, 250 µL of isoflow solution (Beckman Coulter) was added to the cell suspension and incubated for 15 min in dark. Analysis was performed by running the samples on a Navios Flow Cytometer (BD Biosciences, Stockholm, Sweden) and analyzed with Navios analysis software (BD Biosciences).

### 2.6. DNA Isolation and Bisulfite Conversion

Genomic DNA (gDNA) was extracted from PBMCs (n = 23), Jurkat (Jk) and HCV C protein expressing cell lines (JHC.d, JHC.g and JHC.h) using QIAamp DNA minikit (Qiagen). DNA concentration of the samples was measured by Nanodrop and spectrophotometer (Xpose spectrophotometer, Trinean N.V, Gent, Belgium) and quality was assessed by agarose gel electrophoresis. About 200 ng of purified gDNA was bisulfite treated with EZ DNA methylation-Gold kit^Tm^ (Zymo Research, Täby, Sweden) as per the manufacturer’s instruction.

### 2.7. Primer Design, PCR and Generation of Sequencing Library Using Dual Barcoding

The CpG1 island on SHP-1 P2 was selected, and the primers against the bisulfite converted DNA were designed using Applied Biosystems Methyl Primer Express software version 1.0 (Thermo Fisher Scientific). The graphical representation for sequencing library generation was presented in Figure 1. The following three steps were used for constructing the sequencing library for bisulfite-based amplicon sequencing. Step 1 (Inner PCR): After the bisulfite conversion, the region of interest was amplified from the gDNA by using the target specific fwd_primer (5′-GGGTTGTGGTGAGAAATTAATT-3′) and rev_primer (5′-CACACTCCAAACCCAAATAATA-3′). Illumina-specific adapter primer sequences 5′-ACACTCTTTCCCTACACGACGCTCTTCCGATCT-NNNNN-3′ and 5′-AGACGTGTGCTCTTCCGATCT-3′ were attached to the target specific forward and reverse primers, respectively. The five random nucleotides NNNNN in the adapter sequence were added to improve the cluster generation during sequencing. The adapter sequences are part of the sequencing primers and also needed for the hybridization of the amplicon to the surface of flow cell as well as for cluster generation by bridge amplification [37]. To reduce the unspecific binding and primer dimer formation in the PCR reaction, the target was amplified by using HotStarTaq DNA polymerase (Qiagen) active only at high temperature (95 °C). The following PCR conditions were used: 95 °C for 10 min, 35× (94 °C 30 s, 55 °C 30 s, 72 °C 1 min) and 72 °C for 5 min. The amplicon product after first PCR was tested for accurate size and primer dimer formation on 2% agarose gel. Step 2 (Outer PCR): In this step, the amplicon from the first PCR was barcoded with unique dual indexes (tagged with Illumina adapters) at both ends of the DNA to be able to recognize the samples after sequencing multiple samples at the same time. The following outer, frw_primers 5′-AATGATACGGCGACCACCGAGATCTACACi7ACACTCTTTCCCTACACGACG-3′ and rev_primer 5′-CAAGCAGAAGACGGCATACGAGATi5GTGACTGGAGTTCAGACGTGTGCTCTTCCGATCT-3′ were used, where i5 and i7 are an Illumina specific dual indexes [38]. The 8 nucleotide long dual indexes are unique and could be used in combination to sequence a large number of samples in a single run. For more detailed information about the Illumina-specific sequences and protocol, see https://github.com/EnvGen/labProtocols (accessed on 11 February 2019). The outer PCR was performed under the following conditions: 95 °C for 10 min, 10× (94 °C 30 s, 55 °C 30 s, 72 °C 1 min) and 72 °C for 5 min. The quality of PCR products was tested on 2% agarose gel electrophoresis. Step 3 (Clean up): The amplicon product was treated with EXOSAP-IT (Affymetrix) at 37 °C for 15 min, 85 °C for 5 min.

### 2.8. Next-Generation Sequencing (NGS) and Data Analysis

The next-generation sequencing was performed at Clinical Genomics Uppsala (Science for Life Laboratory, Uppsala, Sweden). After PCR cleanup, the DNA concentration of individual libraries was measured using Qubit, and the fragment size was confirmed with Agilent 2200 TapeStation system (Agilent Technologies, Santa Clara, CA, USA) using high-sensitivity D1000 Screen Tape. The libraries were diluted with EB buffer and denatured. Paired-end sequencing was performed on Miseq (Illumina, Inc., San Diego, CA, USA) using the MiSeq^®^Reagent Nano Kit v2. The bisulfite-sequencing data were analyzed by using the CLC genomic workbench software (Qiagen, v.22.0.1). The adapter and indexed sequences were removed from the sequencing reads by using Local Run Manager. The trimmed reads were aligned to the in silico generated bisulfite reference. The methylation call at the CpG site was analyzed by basic variant detection. The intrinsic background noise for each nucleotide was determined separately by analyzing the average error rate at non-CpG sites.

### 2.9. Analysis of Methylation Heterogeneity

The sequencing reads were aligned with bisulfite specific reference sequence using Unipro UGENE (v.43.0). The number of CpG methylations per read for forward and reverse primer was calculated by generating the multiple distance matrix. The methylation percentages of individual reads were plotted as the ratio of analyzed reads versus the number of total methylations per read.

## 3. Results

### 3.1. Quantification of Positive (+) and Negative (−) Strand HCV RNA in Patient-Derived PBMCs

The presence of HCV RNA in PBMCs derived from healthy controls (n = 11) and HCV positive (HCV^+^) patients (n = 12) were detected by two methods: ssqRT-PCR and Abbott real-time HCV assay. As inclusion criteria, patients were selected prior to the start of antiviral treatment against HCV and had not experienced any previous antiviral regimen. HCV targets the liver as a primary site of infection, but the past reports have shown that it also infects the PBMCs to a lesser extent [39,40]. The HCV infection of PBMC could be a reservoir for the relapse after treatment and a source of resistance to the antiviral treatment [41,42]. Furthermore, testing the PBMCs for the specific detection of (+) and (−) strands of HCV RNA will shed light on the infection status as the (−) strand is formed only during the virus replication step in an ongoing infection. The PBMC cell count ranged from 1.4 to 3.2 million cells in healthy controls, whereas in the HCV^+^ patients, the cell count ranged from 1.43 to 4.9 million cells (Table 1).

To further investigate the replicative capacity of HCV, the PBMCs from healthy controls and HCV^+^ patients were analyzed for the presence of (−) and (+) sense HCV RNA strands by strand-specific RTqPCR (ssqRT-PCR). In comparison, the viral load in plasma at a given occasion is also shown in Table 1. As expected, the PBMCs from the healthy controls were negative for the presence of HCV RNA of both polarities. For the HCV^+^ patients, (+) sense HCV RNA was detected in nine of twelve samples (9/12), while no (0/12) (−) sense RNA was detected in any of the samples. The cycle threshold (Ct) values detected for HCV (+) sense RNA in PBMCs from infected patients were above 35 and hence below the quantitative range. In parallel, PBMCs from healthy and HCV-infected individuals were run on Abbott Realtime HCV m2000 assay, which does not discriminate between (+) and (−) polarity. While no viral RNA was detected in the healthy controls (0/11), a low but quantifiable level of viral RNA was detected in all (12/12) HCV infected samples. Low viral titers in PBMCs in commercial assay are consistent with the high Ct values for positive HCV RNA in the ssRT-qPCR assay.

### 3.2. Cell Type Analysis of HCV^+^ PBMCs

**Figure 2:**
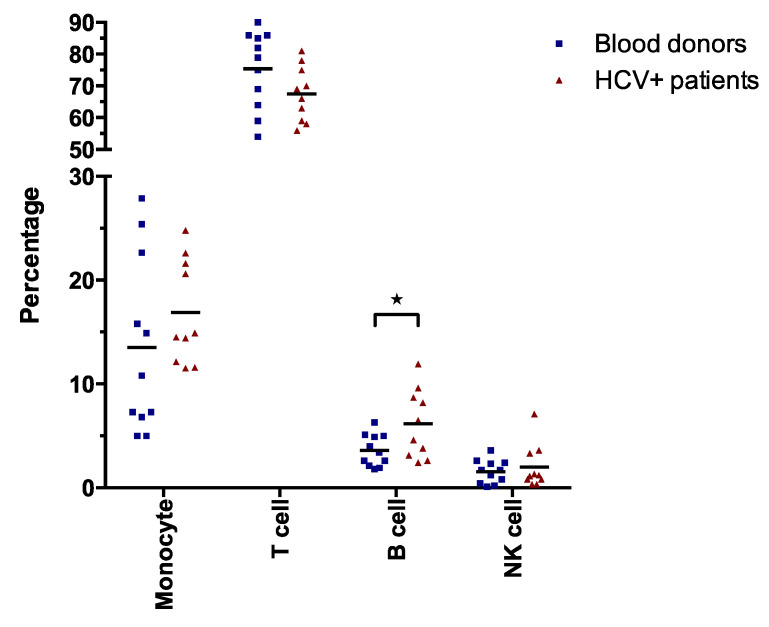
Phenotypic characterization of PBMCs. PBMCs from blood donors and HCV^+^ patients were stained with labeled antibodies that recognize specific surface markers and analyzed by FACS. For cell typing, the following markers were used: CD45+/CD14+ (monocytes), CD45+/CD2+ (T cells), CD45+/CD19+ (B cells) and CD45+/CD16+/CD56+ (NK cells). Statistical comparison was determined by Student’s unpaired *t* test where significant *p* value is indicated with star; * *p* < 0.05.

To compare the PBMC populations between healthy controls and HCV^+^ patient’s cells, we used flow cytometry. The cells were characterized for monocyte, T cell, B cell and NK cell phenotype by using antibodies against the differentiation markers CD45, CD14, CD2, CD19 and CD16/CD56, respectively (Figure 2). The T cells were the major cell type in the PBMC in both healthy control and HCV^+^ patients. Although a slightly lower T cell population was detected in the HCV^+^ patients (67%) compared to the healthy control (75%), this difference was not significant. The proportion of NK cells and monocytes was higher in the HCV^+^ patient group compared to the healthy control group. The NK cell proportion in the healthy control was 1.6% compared to HCV^+^ patients and accounted for 2.0%, whereas the monocyte cell proportion in the healthy control and HCV^+^ patients accounted for 14% and 17%, respectively. The difference in NK cells and monocytes between the control and patient group was non-significant and was considered similar to the T cell population. However, the proportion of B cell type was significantly lower in healthy controls (3.3%) compared to the HCV^+^ patients (6.1%). The higher B cell proportion in HCV^+^ patients compared to the healthy controls is consistent with previous findings that chronic HCV infection is associated with the oligoclonal proliferation of B-cells [43,44].

### 3.3. Methylation Status of CpG1 Island on SHP-1 P2 in HCV^+^ Samples

**Figure 3:**
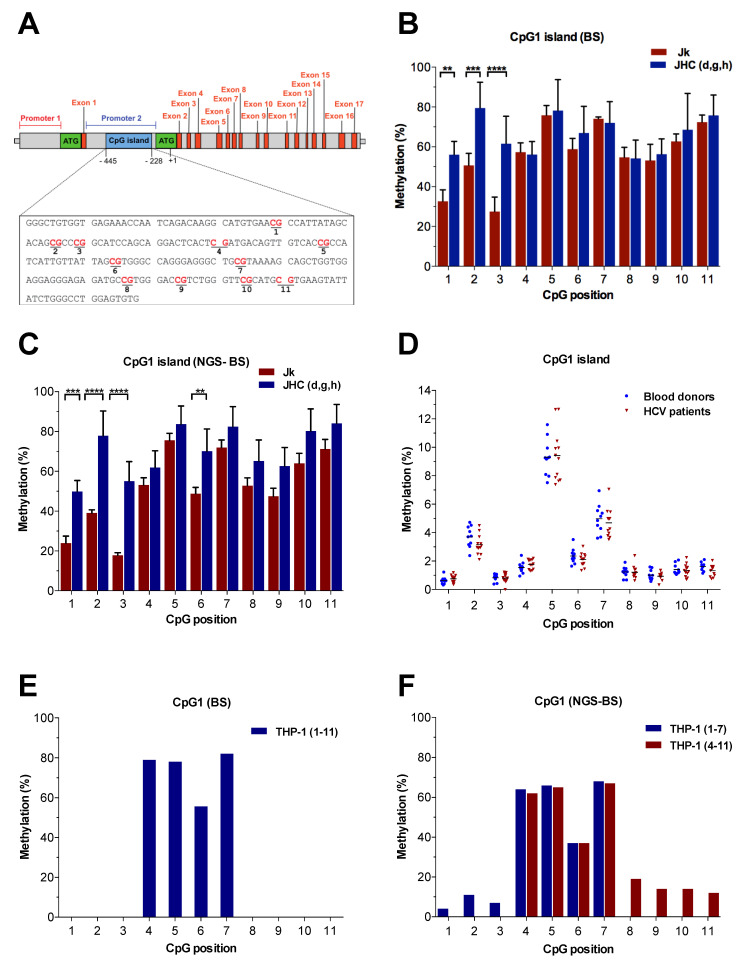
Quantitative CpG methylation pattern on SHP-1 P2 with NGS-BS. (**A**) The SHP-1 gene consisting of 17 exons and two cell-specific promoters, promoter 1 (P1, red horizontal line) and promoter 2 (P2, blue horizontal line). The translation start codons (ATG) for encoded transcripts originating from the P1 and P2 are indicated. The CpG-rich region called CpG island 1 (CpG1, −445 to −228 bp, blue) on SHP-1 P2 was selected for CpG methylation analysis. Individual CpG motifs within the CpG1 island are indicated in red. (**B**) Methylation pattern of SHP-1 P2 CpG1 island in immortalized Jurkat cell lines determined by direct bisulfite sequencing (BS). Data are presented as mean ± SD of two sequencing runs performed in duplicates with both forward and reverse primers. Methylation at individual CpG positions (as indicated in panel (**A**)) within CpG1 island between parental Jurkat (Jk) and HCV C transformed cells (JHC.d, JHC.g and JHC.h). For the C protein expressing cells, data are represented as sum of the averages of JHC.d, JHC.g and JHC.h clones (JHC.d, JHC.g, JHC.h = JHC). The statistical comparison of methylation levels between Jurkat and JHC cells was determined by a two-way ANOVA and Sidak’s multiple comparison test. Bars with stars were statistically significant with adjusted *p* values; ** *p* < 0.01, *** *p* < 0.001, **** *p* < 0.0001. (**C**) Methylation pattern of CpG1 island of SHP-1 P2 promoter in immortalized Jurkat cell lines by NGS-BS. Data are presented as the mean ± SD of two paired-end NGS runs. The statistical comparison of methylation levels between Jurkat and JHC cells was determined as above. (**D**) Methylation pattern of SHP-1 P2 CpG1 island in HCV^+^ PBMCs created with NGS-BS. The gDNA from the PBMC obtained from healthy blood donors (n = 11) and HCV^+^ individuals (n = 12) The DNA methylation at individual CpG motifs in CpG1 island was analyzed, and levels of methylation were compared between healthy blood donors and HCV-infected individuals for each sample. Data are presented as individual dots with mean values indicated. (**E**) Methylation pattern of CpG1 island of SHP-1 P2 promoter in THP-1 cells determined by BS. The presented data are from a single experiment. (**F**) Methylation pattern of CpG1 island of SHP-1 P2 promoter in THP-1 cells by NGS-BS. Data from GpG positions 1 to 7 and 4 to 11 are presented separately.

The methylation of CpG islands on the gene promoter is often associated with the gene silencing [45]. For example, loss or reduced tumor suppressor gene SHP-1 expression due to promoter methylation is common in different types of hematological malignancies [28,29,30,46,47,48]. Therefore, the CpG1 island on SHP-1 promoter 2 (P2) was used as a model promoter to analyze the DNA methylation in the C protein expressing immortalized cells (Jurkat) and in HCV^+^ patient-derived PBMCs (Figure 3A). Notably, in our previous study, we have shown that the expression of the HCV C protein regulates CpG1 island methylation when assessed with classical, Sanger sequencing of the amplified bisulfite-treated DNA (BS-seq) [49]. To analyze SHP-1 CpG methylation, we modified a method that combines the bisulfite (BS) conversion and target amplification by PCR with next-generation sequencing (NGS-BS). To validate the NGS-BS protocol, the Jurkat cells and three individual C proteins expressing Jurkat (JHC.d, JHC.g and JHC.h, collectively as JHC(d,g,h)) cell lines were used as the cell models for CpG1 methylation analysis. Similar to our previous data from Sanger sequencing (Figure 3B) [49], the CpG1 island was methylated at all (11/11) individual CpG motifs in control Jurkat and the C protein expressing cells (Figure 3C). Although each CpG motif in the CpG1 island was methylated in all the cell lines, significant differences in methylation at particular CpG positions were observed. Similarly to the BS-seq, the NGS-BS revealed that the CpG methylation levels between Jurkat and the C expressing clones at CpG1 island were non-significant at positions 4 to 5 and 7 to 11. However, the CpG motifs 1, 2 and 3 were significantly hypermethylated in the JHC (d,g,h) cells compared to the control Jurkat cells by both BS-seq and NGS-BS (Figure 3B,C). Compared to BS-seq, the NGS-BS gave a higher significance value for CpG motifs at position 1 (*** *p* < 0.001 versus ** *p* < 0.01) and position 2 (**** *p* < 0.0001 versus *** *p* < 0.001). In addition, analysis by NGS-BS also showed significant hypermethylation of the CpG motif at position 6 (** *p* < 0.01).

Since the CpG1 island was differentially methylated in the Jurkat and JHC (d,g,h) cells using NGS-BS (Figure 3C), we extended our methylation studies to HCV^+^ clinical samples (Table 1). The PBMCs obtained from the healthy blood donors (n = 11) and HCV^+^ patients (n = 12) were bisulfite-treated and analyzed for methylation of the CpG1 island on SHP-1 P2 by NGS-BS. Although there was a high level of methylation in the CpG1 island of the immortalized cell lines (Figure 3C), there was clearly a much lower level of methylation in the healthy and HCV^+^ PBMCs (Figure 3D). The CpG motifs at position 2, 5 and 7 in both healthy and HCV^+^ PBMCs had the highest methylation signal, corresponding to ≥3%, ≥10% and ≥5%, respectively. In contrast, all the other eight CpG motifs in the CpG1 island had methylation levels below 3% in healthy controls and HCV^+^ samples. Notably, no significant difference in the CpG methylation at CpG1 was detected between healthy controls and HCV^+^ patient-derived PBMCs (Figure 3D).

To further validate the performance of the NGS-BS protocol, we also analyzed the methylation pattern of the CpG 1 island in the monocytic THP-1 cell line. Whereas methylations of CpG positions 4 to 7 were clearly detected by both approaches (Figure 3E,F), the NGS-BS protocol also revealed additional methylations at CpG positions 1 to 3 and 8 to 11 (Figure 3F). In contrast to the high methylation levels found at CpG positions 4 to 7 (37 to 82%), the methylation frequencies at CpG positions 1 to 3 and 8 to 11 were significantly lower (4 to 14%), indicating that the NGS-BS protocol could resolve methylations that were below the detection limit of the BS protocol. Whereas it required peak heights with signal ratios of 20% or higher to reproducibly resolve CpG methylations with the BS protocol, the intrinsic background noise for each base by the NGS-BS protocol was 0.2% or lower (**A**, 0.05%, SD 0.02; **C**, 0.2%, SD 0.04; **G**, 0.1%, SD, 0.04; **T**, 0.07%, SD, 0.01).

### 3.4. Methylation Heterogeneity in Cell Lines

Since the methylation at individual cytosines in a single cell is either methylated or unmethylated, a partial methylation pattern observed at the population level could either be due to the mixture of two distinct cell populations with different methylation status or reflect a heterogeneous methylation status in all cells in the sample. One of the advantages of NGS-BS over direct sequencing of bisulfite-treated DNA is that the methylation status at individual CpG sites could be determined at the allelic level due to the existence of the single nucleotide polymorphisms (SNPs) and that strand-specific differences can be resolved.

**Figure 4:**
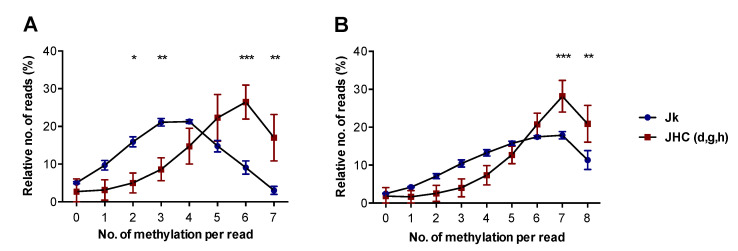
Assessment of methylation heterogeneity of CpG1 island on SHP-1 P2 in cell lines by NGS-BS. The methylation percentages of individual reads for Jurkat (Jk) and JHC cell lines were plotted as ratio of analyzed reads versus number of total methylations per read. (**A**) Relative number of bisulfite sequencing reads per number of methylations generated by forward primer corresponding to CpG site 1 to 7. (**B**) Relative number of bisulfite sequencing reads per number of methylations generated by reverse primer corresponding to CpG site 4 to 11. Data are presented as mean ± SD of two paired-end NGS runs. For the C protein expressing cells, data are represented as sum of the averages of JHC.d, JHC.g and JHC.h clones (JHC.d, JHC.g, JHC.h = JHC)**.** The statistical comparisons of methylation levels were performed by two-way ANOVA and Sidak’s multiple comparison test. Points with stars are statistically significant with adjusted *p* values; * *p* < 0.05, ** *p* < 0.01 and *** *p* < 0.001.

Methylation heterogeneity occurs during embryonic development, epigenetic reprogramming and during cancer, which may associate with gene regulation [50]. In the present study, the allelic heterogeneity at individual CpG sites in Jurkat and the C protein expressing clones was determined by analyses of individual bisulfite sequencing reads. To characterize the allelic heterogeneity in the cell lines, we examined the CpG1 island on the SHP-1 P2 promoter by counting the number of CpG methylations in single bisulfite sequencing reads that contained seven CpG motifs corresponding to CpG position 1 to 7 when sequenced with forward specific primer (Figure 4A) and eight CpG motifs corresponding to CpG position 4 to 11 when sequenced with reverse specific primer (Figure 4B). For each sample, 17,000 to 25,000 reads were obtained, and to enable comparison between the samples, the relative number for each number of methylations was determined. For both conditions, clearly identifiable single peaks that were skewed to the right for the JHC cell lines were identified (Figure 4A,B). For CpG position 1 to 7, Jurkat cells displayed a maximum at three to four methylations and had significantly more reads with two and three methylations, whereas the JHC cell lines displayed a maximum at six methylations and had significantly more reads with six and seven methylations (Figure 4A). For CpG position 4 to 11, the maximum reads for both Jurkat and the JHC cell lines were seven methylations, although the general pattern for the JHC cell lines was skewed to more methylations and had significantly more reads with seven and eight methylations (Figure 4B).

Collectively, our data from the heterogeneity analysis are in line with our findings that the CpG positions 1 to 3 are hypermethylated in the JHC cell lines and also indicates that the positions 4 to 11 when taken together have a higher methylation of CpG motifs in the JHC cell lines compared to Jurkat cells.

## 4. Discussion

In this study, we have established a novel method for the analysis of CpG methylation by NGS. As a working model and for demonstrating proof-of-concept, we chose the CpG1 site of the SHP-1 P2 promoter and comparisons of three different cell systems; immortalized T cells (Jurkat cells and derivatives thereof expressing the HCV C protein), clinical samples (PBMCs from chronic HCV carriers and healthy blood donors) and THP-1; respectively. First, we show that the SHP-1 P2 CpG1 island is surprisingly highly methylated in both control and HCV C expressing T cells. Furthermore, a significantly higher level of CpG methylation was detected at three particular CpG motifs in the C protein expressing cell lines compared to the parental T cells (Figure 3C). This finding was in line with previous results obtained by Sanger sequencing (Figure 3B) [49]. By analysis of the methylation heterogeneity of single reads, we also found that the HCV C expressing cell lines were significantly hypermethylated over the entire CpG1 site, even if the CpG1 sites 1 to 3 were not considered (Figure 4A,B). In contrast, a very low level of methylation at the CpG1 island in the healthy blood donors and HCV-infected patient-derived PBMCs was observed (Figure 3D). One possible explanation for this discrepancy is the heterogenicity of the PBMCs, which consists of functionally distinct cell types (Figure 2). In line with this is a study whereby individual genes may have opposite methylation patterns in different blood cell types, including T cells, B cells, and NK cells [51]. Alternatively, the DNMTs might be upregulated in the immortalized Jurkat cells, which can cause a high level of SHP-1 CpG1 methylation. Truly, the expression of DNA methyltransferases DNMT1, DNMT3B and DNMT3A is often upregulated in different cancer cell lines and tissues, thereby causing the CpG islands hypermethylation of various tumor suppressor genes [52]. The PBMCs used in our study were taken from chronic carriers without detectable (-) RNA strand accumulation (Table 1). This observation indicates that the isolated PBMCs lack active HCV replication and accordingly the C protein expression. Hence, the absence of enhanced CpG methylation (position 1–3, Figure 3D) in PBMCs can be due to the very low C protein expression in this cell population. The cell transformation is a slow and late event which takes years after the initial HCV infection. Previous studies have shown that the methylation of SHP-1 gene promoter and loss of its expression is related to the clinical stages and aggressiveness of the tumor, respectively [30,47]. Therefore, using the cells from the patients with progressive chronic HCV infection developing hepatocellular carcinoma or diffuse large B cell lymphomas could better mimic the SHP-1 methylation status seen in Jurkat cells. Alternatively, the epigenetic effects of HCV on the SHP-1 P2 promoter might be fundamentally different in Jurkat cells and PBMCs.

Whereas CpG methylation in cell lines and clinical samples often is an all or none phenomenon, where most of the CpG sites are either fully methylated or not methylated at all, CpG methylation of the SHP-1 P2 promoter in Jurkat cells displays a more heterogenous pattern where most of the CpG sites are hemi-methylated [28,30,31,32,47,49,53]. Since the methylations of individual cytosines in a single cell are either methylated or unmethylated, the partial methylation pattern observed could either be due to the mixture of two distinct cell populations with different methylation status or reflect a heterogeneous methylation status in the entire cell population. Although we cannot exclude any cis-acting effects on epigenetic regulation in Jurkat cells, we were nevertheless unable to find any indications for two separate populations and thus favor a model with a continuous distribution of the CpG methylations (Figure 4A,B).

A similar approach for the quantification of locus-specific CpG methylation by next-generation sequencing was used by Masser et al. and Bashtrykov et al. [33,37]. However, our method differs from the aforementioned methods in the preparation of sequencing library. Masser et al. have used a tagmentation approach, while Bashtrykov and colleagues used a ligation-based method, which is challenging and requires more optimization. Our PCR-based approach for generating the sequencing library (Figure 1) is cost-effective and relatively simple. The possibility of PCR-based biases is minimized by carefully optimizing the primers and cycling conditions and also using fewer PCR cycles in outer PCR (10 cycles). The method also provides the advantage of multiplexing by pooling multiple libraries that can be sequenced in a single sequencing run. In multiplexed libraries, each sample is given a separate identity by adding unique indexes (eight nucleotides, see Materials and Methods) to the both ends of the DNA template during the library preparation (Figure 1).

## 5. Conclusions

Collectively, the described next generation sequencing of bisulfite amplicon is a sensitive and cost-saving method and can be used to quantitatively characterize the CpG methylation status at the level of individual CpG position in cell lines, virus-associated and non-associated tumor samples. Compared to conventional BS-sequencing, NGS-BS not only offers higher precision and higher sensitivity but also allows assessment of cis-acting effects of epigenetic regulation.

## Figures and Tables

**Table 1:** Detection of total and strand specific (+ and −) HCV RNA in PBMCs from HCV^+^ patients.

Sample ID	Cell Count (10^6/mL)	ssqRT-PCR HCV (+) RNA	ssqRT-PCR HCV (−) RNA	Abott Real-Time HCV Assay (IU/10^6 cells)	Viral Load Plasma (IU/mL)
615062	2.46	Positive	ND	2900	2,700,000
615063	2.64	Positive	ND	1600	5,000,000
615064	1.84	ND	ND	53	260,000
615067	3.2	Positive	ND	1100	9,100,000
615068	3.9	Positive	ND	160	1,800,000
615069	2.3	ND	ND	29	610,000
615070	2.2	Positive	ND	840	230,000
615071	2.65	Positive	ND	900	240,000
615072	4.9	Positive	ND	490	1,900,000
615073	1.44	Positive	ND	2000	3,300,000
615074	1.43	ND	ND	9	1500
615075	1.7	Positive	ND	72	1,500,000

ND: not detected.

## Data Availability

Raw data from the experiments are available upon request.
